# Update on Dendritic Cell-Induced Immunological and Clinical Tolerance

**DOI:** 10.3389/fimmu.2017.01514

**Published:** 2017-11-20

**Authors:** Carolina Obregon, Rajesh Kumar, Manuel Antonio Pascual, Giuseppe Vassalli, Déla Golshayan

**Affiliations:** ^1^Department of Medicine, Transplantation Centre and Transplantation Immunopathology Laboratory, Service of Immunology, Centre Hospitalier Universitaire Vaudois, University of Lausanne, Lausanne, Switzerland; ^2^Department of Surgery, Transplantation Centre, Centre Hospitalier Universitaire Vaudois, University of Lausanne, Lausanne, Switzerland; ^3^Département coeur-vaisseaux, Centre Hospitalier Universitaire Vaudois, Lausanne, Switzerland; ^4^Fondazione Cardiocentro Ticino, Swiss Institute of Regenerative Medicine (SIRM), Lugano, Switzerland

**Keywords:** tolerogenic dendritic cells, autoimmune diseases, immunotherapy, solid organ transplantation, tolerance

## Abstract

Dendritic cells (DCs) as highly efficient antigen-presenting cells are at the interface of innate and adaptive immunity. As such, they are key mediators of immunity and antigen-specific immune tolerance. Due to their functional specialization, research efforts have focused on the characterization of DCs subsets involved in the initiation of immunogenic responses and in the maintenance of tissue homeostasis. Tolerogenic DCs (tolDCs)-based therapies have been designed as promising strategies to prevent and control autoimmune diseases as well as allograft rejection after solid organ transplantation (SOT). Despite successful experimental studies and ongoing phase I/II clinical trials using autologous tolDCs in patients with type 1 diabetes, rheumatoid arthritis, multiple sclerosis, and in SOT recipients, additional basic research will be required to determine the optimal DC subset(s) and conditioning regimens for tolDCs-based treatments *in vivo*. In this review, we discuss the characteristics of human DCs and recent advances in their classification, as well as the role of DCs in immune regulation and their susceptibility to *in vitro* or *in vivo* manipulation for the development of tolerogenic therapies, with a focus on the potential of tolDCs for the treatment of autoimmune diseases and the prevention of allograft rejection after SOT.

## Introduction

Dendritic cells (DCs) are at the interface of innate and adaptive immunity and, thus, are key mediators of immunity and tolerance. Importantly, DCs constitute a heterogeneous population that comprises multiple subsets exhibiting distinct functional specializations that vary according to their origin, maturation state, location, and environmental conditions (1, 2). In their immature state, DCs mainly traffic and reside in peripheral tissues where they can capture antigens and process them into major histocompatibility complex (MHC):peptide complexes. DCs undergo maturation not only after microbial infection but also in response to damage-associated molecular patterns (DAMPs) and pro-inflammatory cytokines produced as a result of tissue injury. This is the cornerstone for the initiation of effective adaptive immune responses (3, 4). Extensive experimental data over the years have highlighted the possibility of generating “tolerogenic DCs” (tolDCs) that are maturation-resistant *in vitro*, express low levels of T-cell costimulatory molecules, and a have a reduced capacity to produce pro-inflammatory cytokines. These tolDCs mediate antigen-specific T-cell hyporesponsiveness and promote the expansion and/or induction of regulatory T cells (Treg). Thus, the potential for tolDCs to dampen immune responses may be used clinically, e.g., in autoimmune diseases and after solid organ transplantation (SOT). In this review, we briefly describe human DC subsets and the immune regulatory mechanisms mediated by these cells. We then discuss how DCs may be manipulated in the perspective of tolerogenic immune therapies.

## Human DC Subsets and Functional Specialization

Dendritic cells represent a heterogeneous cell population arising from bone marrow-restricted precursors identified in mice and humans (5). In humans, common DC progenitors give rise to plasmacytoid DCs (pDCs) and intermediate precursors of conventional DCs (pre-cDCs) that are pre-committed to become either CD1c^+^ (BDCA-1) or CD141^+^ (BDCA-3) conventional DCs (cDCs) (6). The HLA-DR^+^CD14^−^CD11b^−^ fraction of human peripheral blood mononuclear cells (PBMCs) comprises the CD1c^+^ DC subset (characterized by CD172α and IRF4 expression), the CD141^high^ DC subset (characterized by Clec9A, XCR1, IRF8, and TLR3 expression), and the pDC subset (identified by BDCA-2, BDCA-4, and CD123^bright^ expression) (Table 1).

**Table 1:** Characteristics of blood human dendritic cells (DCs) and monocyte-derived DCs (ModDCs) subsets.

	CD1c	CD141	Plasmacytoid DCs (pDCs)	ModDCs	Reference
Surface expression, intracellular markers or transcriptional markers	Lin^−^, MHC II^+^ CD1c^+^ (BDCA-1^+^) CD11c^hi^, CD11b^−^ CD4^+^, CD2^+^ CD45RO^+^ CD172α^+^ IRF4^+^	Lin^−^, MHC II^+^ CD141^hi^ (BDCA-3^hi^/Thrombomodulin) CD11c^+^,CD11b^−^ CD4^+^, CD2^−^ TLR3^+^ Clec9A^+^ (DNGR-1^+^) XCR1^+^ IRF8^+^	Lin^−^, MHC II^+^ CD303^+^ (BDCA-2^+^) CD304^+^ (BDCA-4^+^/Neuropilin-1) CD11c^−^, CD11b^−^ CD123^+^ CD45RA CD4^+^ ILT7^+^ TLR7^+^, TLR9^+^	CD1c^+^ (BDCA-1^+^) CD14^+^ CD11c^+^, CD11b^+^ DC-SIGN^+^Additional markers expressed in tissue ModDCs:CD1a^+^ CD206^+^ FcεRI^+^ FLT3^+^ IRF4^+^ Zbtb46^+^	(6–17)
Frequency in peripheral blood(% peripheral blood mononuclear cell)	0.2 ± 0.1%	0.02 ± 0.01%	0.2 ± 0.1%	≈0.29 ± 0.2%	(18, 19)
Functional specialization	Excel in CD4^+^ T-cell priming. Th1 and Th17 polarization.	Cross-presentation of soluble antigens to CD8^+^ T cells. Secretion of Type-I IFN (poly I:C)	Type-I IFN secretion in response to viral infections. Liver and respiratory tract pDCs promote tolerance.	Naïve and memory CD4^+^ T-cell stimulation. Th17 polarization. DC-10, skin CD141^+^CD14^+^ DCs and CD1c^+^CD14^+^ DCs in melanoma patients promote tolerance.	(6, 9, 20, 21)
Mouse equivalent	CD4^+^, CD11b^+^ (lymphoids) CD11b^+^ (tissues) CD172α^+^ IRF4^+^	CD8α DCs (lymphoids) CD103 DCs (tissues) XCR1^+^ IRF8^+^	B220 Siglec H BST2 (PDCA1)	Ly6C^+^ CD172α^+^ DC-SIGN^+^ CD206^+^ FcεRI^+^	(7, 22, 23)

*BDCA, blood dendritic cells antigen; DNGR-1, dendritic cell natural killer lectin group receptor-1; IFN, interferon; ILT, immunoglobulin-like transcript; Lin, lineage; MHC II, major histocompatibilty complex class II; TLR, toll-like receptor*.

### Peripheral Blood DCs

Peripheral blood DCs are likely the precursors of DCs found in peripheral tissues and lymphoid organs. In peripheral tissues, there is evidence for high phenotypic heterogeneity of DCs, contrasting with the well-defined phenotypic expression of blood DCs. In addition to the BDCA population and Langerhans cells, other subpopulations of tissue DCs can be distinguished by the expression of langerin, CD1a, and CD14 (24, 25); however, these markers are promiscuously expressed making it difficult to unambiguously discriminate peripheral tissue DC subpopulations (24). For example, studies in patients with allergic asthma show that most of the lung CD1c^+^ (BDCA-1^+^) DCs also express CD141 (BDCA-3) (26). Recently, using gene expression profiling and mass cytometry analysis, a set of lineage-imprinted cell-surface markers, such as CD172α/IRF4 and XCR1/IRF8, were identified, allowing for a better discrimination between CD1c^+^CD14^−^ DC and CD141^+^CD14^−^ DC subsets in human tissues (7).

### Lymphoid Organs DCs

Multiple subsets of DCs have been found in lymphoid organs; however the distinction between migratory and lymphoid organs-resident DCs still requires further investigation. CD1c^+^CD14^−^ DC, CD141^+^CD14^−^ DC, and pDC subsets have been found in the human spleen, tonsils, and axillary and pulmonary lymph nodes (LNs) (8–10, 27). These subsets likely correspond to the resident DC population. In axillary LNs, pDCs have been reported to localize in the paracortex (27). The CD141^+^CD14^−^ DC subset, characterized by Clec9A expression, is mostly distributed around the LN cortex (inner and outer) (25). By contrast, CD1c^+^CD14^−^ DCs have been reported to localize within the T-cell zone in close proximity to the B-cell zone (10). Interestingly, in axillary and pulmonary LNs, but not in the spleen and tonsils, a high frequency of a HLA-DR^+^ cells with the CD141^+^CD14^+^DC-SIGN^+^CD206^+^CD1c^+^CCR7^int/low^ phenotype has been reported, suggesting that this subset could be related to a migratory subset. This population is found in the diffuse T-lymphocyte regions of the LN paracortex (10, 27).

### Monocyte-Derived DCs (ModDCs)

*In vitro* experiments have documented that monocytes are important precursors of DCs (28, 29). However, it has been difficult to properly identify ModDCs *in vivo* due to common features shared by cDCs, monocytes and macrophages. Recent data suggest that a ModDCs subset may exist in humans (10–12, 25, 30). For example, studies in steady-state conditions described a subpopulation of cells expressing CD1c^+^CD14^+^HLA-DR^+^ in both blood and bronchoalveolar lavage fluid (BALF) (10, 18). Although it was demonstrated that blood CD1c^+^CD14^+^ cells have monocytic features, these cells have increased antigen-presenting ability and a different gene signature compared to monocytes (18). Interestingly, in non-diseased lung tissue CD1c^+^CD14^+^ populations were shown to be enriched for the gene signatures of ModDCs described in the literature, which includes the expression of *ZBTB46, IRF4*, and *FLT3* genes (10). During inflammation, CD1c^+^CD14^+^ cells have been reported in the BALF from sarcoidosis patients co-expressing CD141, CD123, and DC-SIGN, or in synovial fluid from rheumatoid arthritis (RA) patients and carcinomatous ascites from untreated cancer patients co-expressing CD1a, FcεRI, CD172a, and CD206 (11, 12). These cells were enriched for the ModDC signature and functionally ModDC from ascites showed an important capacity to polarize naive T cells into Th17 cells as well as to stimulate memory CD4 T cells to produce IL-17 (11).

In the past few years, additional DC subsets were associated with the induction of immune tolerance; however, their precise ontogeny and phenotype remains to be fully established. Gregory and co-workers described a DC subset expressing HLA-DR^+^CD14^+^CD16^+^ receptors in human blood, which was able to induce type 1 regulatory T (Tr1) cells through the release of IL-10; hence, its name DC-10 (31). Furthermore, the presence of a DC subset expressing HLA-DR^+^CD141^+^CD14^+^ was reported in skin dermis. This subset exhibited a potent inhibitory activity on skin inflammation.

### Functional Specialization of DCs

In terms of function, DCs can exhibit an immature phenotype at steady-state or a mature phenotype upon exposure to inflammatory stimuli. Immature DCs have a unique immune surveillance function. At this stage, DCs express low levels of MHC and costimulatory molecules such as CD80/B7.1, CD86/B7.2, CD40, OX40L, inducible T-cell costimulatory ligand, as well as low expression of adhesion molecules such as intercellular adhesion molecule-1 (ICAM-1/CD54) (32). Interestingly, at steady-state tissue CD1c^+^CD14^−^ DCs exhibit a higher activation state, e.g., higher expression levels of CD80, CD83, CD86, and CD40 compared with their blood counterparts (22, 30).

Quiescent immature DCs can mature and become activated in local tissues in the presence of pathogen-associated molecular patterns or DAMPs in the context of sterile injury (e.g., autoimmunity or ischemia/reperfusion) and local inflammatory mediators (IFN-α, IL-1β, IL-6, TNF-α, or CD40L/CD154). Within the context of this maturation process, DC function is regulated by a core set of genes controlled by NF-κB and IFN-mediated signaling (33). In this process, immature DCs evolve from an antigen-capturing mode to an antigen-processing and antigen-presenting mode by upregulating MHC molecules and costimulatory molecules along with chemokine receptors. This allows them to migrate to specialized lymphoid organs, release the corresponding polarizing cytokines, and initiate specific adaptive immune responses.

Regarding the fate and function of human DCs, both unstimulated CD1c^+^CD14^−^ and CD141^+^CD14^−^ DCs from blood, non-lymphoid, and lymphoid tissues were shown to be more immunogenic than pDCs, with an increased capacity to process and present soluble foreign antigens, including transplant-derived alloantigens, as immunogenic MHC:peptide complexes to CD4^+^ T cells (25, 34–36). It has been reported that both blood CD1c^+^ DCs and CD141^+^ DCs efficiently induce Th1 polarization in allogeneic co-culture assays, the latter with increased release of IFN-γ upon maturation (9). CD141^+^ DCs were also shown to be more efficient at inducing Th2 cells compared to CD1c^+^ DCs (20). By contrast, both CD1c^+^ and CD141^+^ DCs derived from lymphoid tissues efficiently induced Th1 and Th2 responses (21). In lung tissues, CD1c^+^ DCs were shown to have a great capacity to induce Th17 responses following *A. fumigatus* challenge (37). In addition to their capacity to induce effector CD4^+^ T cells, all DC subsets isolated from lymphoid tissues were able to efficiently cross-present soluble antigens to CD8^+^ T lymphocytes (21). CD141^+^ DCs are referred to as “human cross-presenting DCs” due to their functional homology with mouse CD8α^+^ DCs (38, 39), in particular with respect to the expression of TLR3 which promotes cross-priming and is required for the production of large amounts of IFN-λ upon TLR3 ligation (13).

In comparison to cDCs, pDCs have a similar distribution in peripheral blood and lymphoid organs, but are present at lower numbers in tissues (30, 40). In the immature state, pDCs express lower levels of costimulatory molecules but multiple pattern recognition receptors that are important for type-I IFN secretion, including intracellular TLR7 and TLR9 (14). In the presence of infectious or inflammatory stimuli, pDCs traffic to lymphoid organs and sites of inflammation. While their role was first described in response to viral infections via the recognition of nucleic acids, tissue-resident gut and airway pDCs have been shown to exert a pivotal role in oral and mucosal tolerance (15, 16, 41). In experimental mouse models of SOT as well as in clinical liver transplantation, pDCs were associated with the generation of alloantigen-specific Treg promoting prolonged allograft survival (42–45). Activated pDCs could also induce CD8^+^ Treg in *in vitro* co-cultures (46).

Overall, these studies highlight the diverse responses of DCs depending on their origin. *Bona fide* cDC were demonstrated to have an inherent capacity to induce immunogenic responses, while pDCs and some immature ModDC subsets participate in tolerance induction. In humans, however, the functional specialization of DCs in the polarization of T cells appears to be less sharply defined compared to mice. The nature and the intensity of the stimuli, as well as the local environment, play an important role in determining the functional specialization of human DCs.

## Mechanisms of Immune Regulation by DCs

Dendritic cells play an important role in the maintenance of immune homeostasis and self-tolerance under steady-state conditions. Their significant role in the induction and maintenance of tolerance has been demonstrated in experimental models. Constitutive or conditional depletion of cDCs was shown to break self-tolerance of CD4^+^ T cells, leading to spontaneous development of lethal autoimmunity manifested by splenomegaly, neutrophilia, autoantibody formation, and an increased frequency of Th1 and Th17 effector cells (47, 48).

### Surface Molecules Expressed on tolDCs

In the absence of local inflammation, DCs remain immature with low surface expression of MHC class II and costimulatory molecules, reflecting their participation in the maintenance of peripheral immune tolerance. Indeed, some DC subsets, such as CD103^+^ DCs in mice and pDCs, blood DC-10, and skin CD141^+^CD14^+^ DCs in human, exhibit inherent tolerogenic properties including the ability to induce Treg and/or promote T-cell hyporesponsivness to antigenic stimuli (15, 31, 49, 50). In addition to low expression of MHC class II and costimulatory molecules, tolDCs overexpress inhibitory molecules such as HLA-G, programmed death ligand (PD-L)-1 and PD-L2, and galectins that contribute to their tolerogenic potential. HLA-G is a non-classical MHC class I antigen that plays an important role in materno-fetal tolerance. Through interactions with inhibitory receptors expressed on maternal NK cells (killer cell immunoglobulin-like receptor, KIR) and T cells (ILT2 and ILT4), the expression of HLA-G on fetal cells protects them against maternal alloreactive cytotoxic cells (51). HLA-G engagement of the human inhibitory receptor ILT4 overexpressed on DCs in transgenic mice promoted long-term survival of allogeneic skin grafts, in part as a result of the downregulation of MHC class II and costimulatory molecules, leading to the induction of Treg and hyporesponsiveness of the alloreactive T-cell repertoire (52). Expression levels of PD-L1 and PD-L2 on DCs increase during DC maturation. These ligands can interact with the inhibitory receptor PD-1 expressed on activated T cells and Treg, thus contributing to T-cell homeostasis (53). Galectins have been identified as important regulators of T cells and DCs (54–56). Galectin-1 was shown to inhibit T-cell effector functions by promoting growth arrest and apoptosis of activated T cells (57, 58), and by blocking pro-inflammatory cytokines secretion by DCs (59). Moreover, galectins are overexpressed in the microenvironment of tumors and have been implicated in their immune escape. In *in vivo* models, DCs constitutively expressing galectin-1 delayed the onset of autoimmune diabetes in mice (60). Conversely, galectin-1-deficient mice experienced accelerated rejection of skin allografts (61).

### Immunomodulatory Molecules Secreted by tolDCs

Tolerogenic DCs were shown to secrete molecules, such as transforming growth factor-beta (TGF-β), IL-10, and indoleamine 2,3-dioxygenase (IDO), which favor a tolerogenic environment and the induction and/or expansion of Treg. TGF-β is a pleiotrophic cytokine involved in multiple cellular functions, including growth, differentiation, proliferation, remodeling, apoptosis, and immune homeostasis. TGF-β is secreted in a latent form complexed with latent TGF-β binding protein and latency-associated peptide. tolDCs were shown to play a crucial role in both the release of TGF-β and activation of the latent TGF-β protein complex (62, 63). TGF-β has been also involved in the induction of Foxp3 expression and peripheral conversion of conventional naïve CD4^+^ T cells into induced Treg (iTreg) in the presence of IL-2 (64, 65). Through their constitutively high expression of the inhibitory receptor cytotoxic T-lymphocyte antigen-4 (CTLA-4/CD152), Foxp3^+^ Treg ligate B7.1/2 expressed on mature DCs and outcompete costimulatory CD28 unregulated on effector T cells (Teff) (66). The interaction between B7.1/2 and CTLA-4 was shown to promote the expression of IDO by DCs, a potent regulatory molecule that catalyzes the degradation of tryptophan required for Teff functions (67–72). In addition, tryptophan catabolites, such as kynurenine, quinolinic acid, and 3-hydroxyanthranilic acid exhibit direct immunosuppressive properties (73, 74).

### Function of tolDCs

Mouse CD103^+^ DCs as well as both human and mouse pDCs were shown to mediate oral tolerance through an IDO-dependent mechanism (49). In human blood, DC-10 induce and expand Tr1 cells through the release of IL-10 and TGF-β (31, 75). DC-10 identified as CD11b^+^CD11c^+^CD14^+^CD16^+^CD83^+^HLA-DR^+^ cells that do not express CD1a and CD1c. Furthermore, DC-10 expresses the inhibitory receptors ILT2, ILT3, ILT4, and HLA-G. Despite concomitant high surface levels of costimulatory molecules (CD40, CD80, and CD86), DC-10 exhibit potent tolerogenic activity (31). Another DC population characterized by CD1c^+^CD14^+^CD16^−^ expression was found in the blood of melanoma patients and exhibited immunosuppressive functions by suppressing T-cell proliferation in an antigen-specific manner. It was suggested that this DC subtype modulated T-cell responses through the expression of PD-L1 (18). A similar phenotype was described in minced lung tissues. The frequency of the CD1c^+^ subset (including the CD1c^+^CD14^+^ fraction) was increased in patients with chronic obstructive pulmonary disease, suggesting that these cells may be involved in the enhanced susceptibility of these patients to infections. Indeed, this subset favored the generation of IL-10-secreting CD4^+^ T cells and mediated immunosuppression through IL-10, IL-27, and ICOS-L (76). In skin dermis, resident CD141^+^CD14^+^ DCs were shown to produce large amounts of IL-10 and were able to induce Treg (50).

Besides their role in controlling peripheral immune responses, DCs play a role in the maintenance of central tolerance, as trafficking peripheral DCs can home to the thymus and promote negative selection of antigen-reactive T cells, thus contributing to a safe peripheral T-cell repertoire (77, 78). By presenting antigens directly within the thymus, DCs and particularly resident pDCs influence the generation of natural Foxp3^+^ Treg, a process mediated by the IL-7-related molecule, thymic stromal lymphopoietin (TSLP), which is secreted in the thymic medulla (79–81).

## Generation of tolDCs

**Figure 1:**
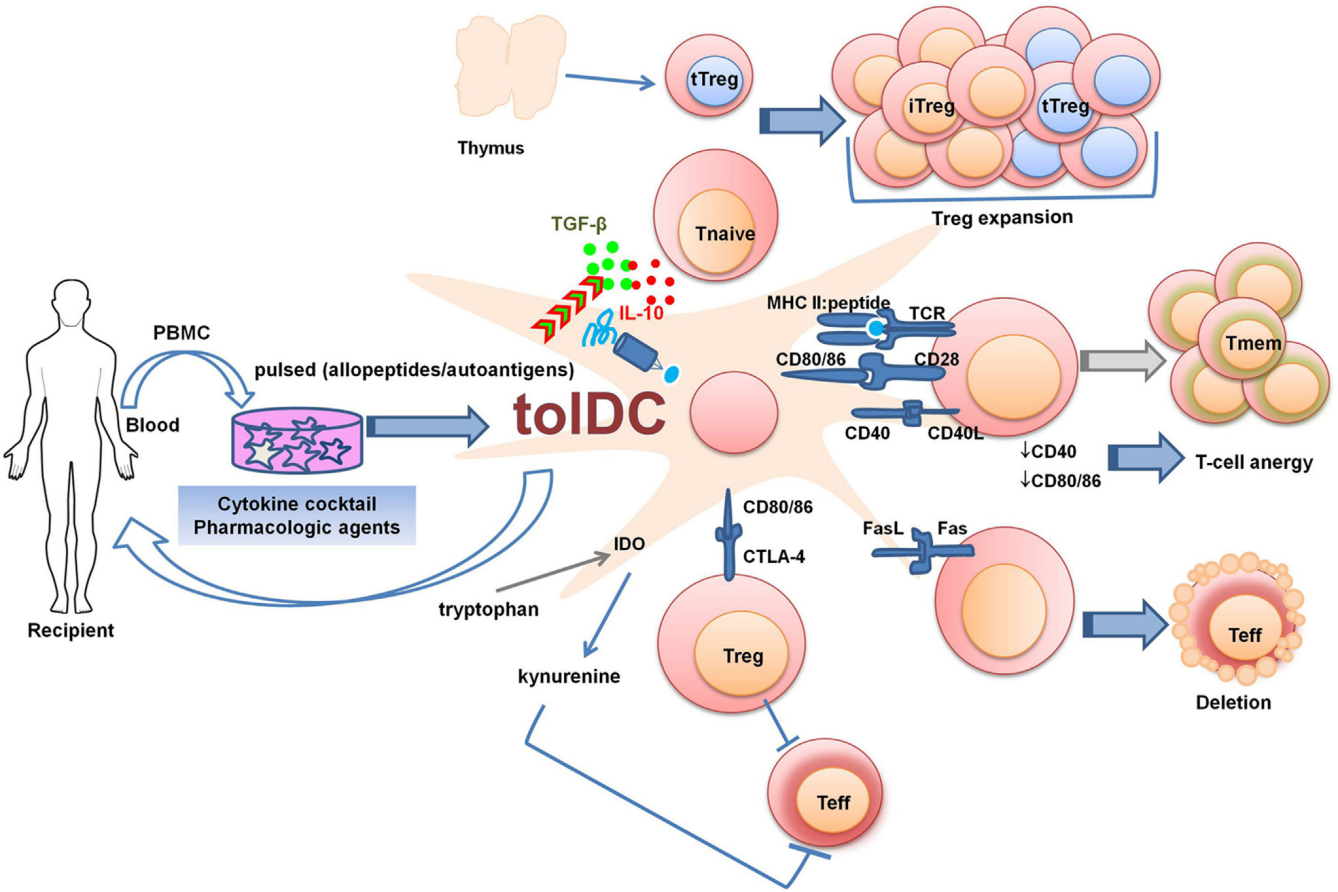
Strategies to generate tolDCs for clinical therapeutics. PBMCs or bone marrow-derived cells can be differentiated into tolDCs in the presence of pharmacologic agents and immunomodulatory cytokines. The generated tolDCs are either donor-derived (in the case of living-donor transplantation) or autologous (in the case of autoimmune diseases or transplantation) and can be further pulsed *in vitro* with specific antigens (peptides, donor cell lysates, apoptotic cells). tolDCs can regulate Teff responses by various mechanisms: 1. Fas/FasL pathway-mediated deletion; 2. Production of IDO which degrades the essential amino acid tryptophan through kynurenine pathway, causing starvation of Teff. The production of IDO is favored by reversed signaling via interaction between CD80/CD86 on DCs and CTLA-4 on regulatory T cell (Treg). 3. Surface expression of inhibitory molecules and secretion of regulatory mediators. Abbreviations: CTLA-4, cytotoxic T-lymphocyte antigen-4; IDO, indoleamine 2,3-dioxygenase; iTreg, induced regulatory T cell; PBMC, peripheral blood mononuclear cells; tolDC, tolerogenic dendritic cell; Teff, effector T cell; Tmem, memory T cell; Tnaive, naïve T cell; tTreg, thymic-derived regulatory T cell.

Dendritic cell-based therapeutic approaches are being explored with the aim to reestablish self-tolerance in autoimmune diseases, and to promote alloimmune tolerance after SOT. Several strategies for the generation of tolDCs are being explored (Figure 1). These include treatment with pharmacologic agents or cocktails of immunomodulatory cytokines, genetic engineering, and exposure to apoptotic cells. Research groups have developed protocols to generate and expand antigen-specific tolDCs *in vitro*. Most of these *in vitro* conditioning regimens aim to stabilize the immature state of DCs, even in the presence of strong inflammatory challenges [e.g., lipopolysaccharide (LPS)]. The resulting tolDCs also express and/or secrete immunomodulatory molecules that favor the development and expansion of Treg (82, 83). After adoptive transfer *in vivo*, maturation-resistant tolDCs may, therefore, promote peripheral tolerance mainly by inducing antigen-specific T-cell hyporesponsiveness and an immuno-regulatory microenvironment.

### Pharmacologic Interventions for tolDC Induction

Various pharmacological agents have been used to generate tolDCs, including immunosuppressive drugs, cyclic AMP inducers, chemicals, cytokines, and growth factors (Table 2) (84, 85). Many of these agents were primarily studied for their inhibitory effects on T-cell activation and proliferation. As such, some of them are currently used in the clinical treatment of autoimmune diseases and in the prevention of allograft rejection after SOT.

**Table 2:** Pharmacologic interventions to induce tolerogenic DCs (tolDCs).

Therapeutic agents	Immunomodulatory substances
Immunosuppressive drugs	CTLA-4 Ig (86)
	Rapamycin (87), Cyclosporine A (88), Tacrolimus (89)
	Mycophenolic acid formulations (90, 91)
	Corticosteroids (92)
	DSG analogs (93, 94)
Cyclic AMP inducers	Prostaglandine E_2_ (95)
	Histamine (96)
Chemicals	Vitamin D3 (91, 97)
	Aspirin (98–100)
	Activator of the aryl hydrocarbon receptor (101)
Proteins and neuropeptides	HLA-G (31, 52)
	C4b-binding protein α7β0 isoform (102)
	Vasoactive intestinal peptide (103–105)
	α-melanocyte-stimulating hormone (106, 107)
Cytokines	Low doses of GM-CSF without IL-4 (108)
	IL-10
	TGF

*AMP, adenosine monophosphate; DSG, 15-deoxyspergualine; GM-CSF, granulocyte-macrophage colony-stimulating factor; HLA, human leukocyte antigen; IL, interleukin; TGF, transforming growth factor-beta*.

Dexamethasone, a potent immunosuppressant, blocks the differentiation and maturation of DCs and enhances their death by apoptosis (92, 109). Using rat bone marrow-derived DCs and human GM-CSF/IL-4-induced ModDCs, we demonstrated that pretreatment with dexamethasone-induced selective expansion of Treg and T-cell alloantigen-specific hyporesponsiveness in re-challenge experiments (110). Dexamethasone was reported to have synergistic effects with other drugs to induce tolDC, in particular 1,25-dihydroxyvitamin D3 (VitD3) (111). While traditionally known for its role in the regulation of calcium and bone homeostasis, VitD3 and its receptor were also described to regulate innate and adaptive immune responses. Exposure to VitD3 inhibited the expression of MHC class II, CD80, and CD86 on DCs with a high ratio of PD-L1/CD86, while reducing the production of pro-inflammatory cytokines, such as IL-12 and IL-23, and increasing that of TGF-β and IL-10. Moreover, VitD3 favored Treg development and blocked B-cell proliferation and differentiation toward antibody-producing plasma cells (112).

Inhibitors of the mammalian target of rapamycine (mTOR) pathway engage FK506-binding protein 12 forming a complex that blocks mTOR, but not calcineurin, resulting in non-specific inhibition of cell cycle progression and, therefore, of T- and B-cell proliferation. *In vitro* assays, together with experimental and clinical data, suggest that immunosuppression based on mTOR-inhibitors may favor the induction of peripheral tolerance. In rodent models, the adoptive transfer of rapamycin-conditioned alloantigen-pulsed DCs resulted in prolonged cardiac and skin allograft survival (87, 113). Moreover, rapamycin was shown to facilitate peripheral deletion of alloreactive Teff by promoting activation-induced cell death in experimental transplantation models, while selectively expanding human Foxp3^+^ Treg and Tr1 cells both *in vitro* and *in vivo* (114–117).

Various other immunosuppressive drugs and biologic agents were described to generate tolDCs *in vitro* including mycophenolic acid (MPA) formulations (90, 118), deoxyspergualin (DSG) and its analogs (93), aspirin (98, 99), retinoic acid (117), and prostaglandin E_2_ (119). These substances mainly interfere with NF-κB pathway-mediated DCs maturation and the capacity of DCs to produce IL-12p70 (84, 85).

Bone marrow-derived DCs exposed *in vitro* to the immunomodulatory cytokines IL-10, TGF-β, or low-dose GM-CSF in the absence of IL-4 exhibit low expression levels of costimulatory molecules and pro-inflammatory cytokines with minor changes in MHC class-I and -II molecules. These conditioned DCs were less immunogenic when co-cultured with CD4^+^ and CD8^+^ T cells, while promoting the expansion of Treg (both natural Treg and iTreg) and antigen-specific T-cell hyporesponsiveness *in vivo* upon re-challenge (108, 120).

Some of these agents also directly promote the differentiation of tolDCs *in vivo*. For example, local or systemic presence of cytokines such as IL-10, TGF-β, and even IFN-γ-induced differentiation of monocytes into tolDCs and promoted FoxP3^+^ Treg (121). The eye is a known locally immune-privileged site and the aqueous humor constitutively contains molecules that maintain DCs in an immature state, such as TGF-β2, IDO, the neuropeptide α-melanocyte stimulating hormone (α-MSH) and FasL (122). The epithelium also plays a critical role in dampening inflammation through the release of epithelial-derived factors, including prostaglandin E_2_, TSLP, retinoic acid, and TGF-β, which are able to promote tolDC (119, 123).

Cobalt protoporphyrin is an inducer of heme oxygenase (HO)-1, an intracellular enzyme that catalyzes the degradation of heme, resulting in the production of biliverdin and carbon monoxide. HO-1 expression is induced by local oxidative stress. Experimentally, HO-1 upregulation was protective in the context of inflammatory processes and after allogeneic SOT (124). Besides maintaining HO-1 expression on human DC, cobalt protoporphyrin prevents their maturation and promotes the secretion of regulatory cytokines (124, 125). Interestingly, HO-1 is also able to inhibit the activation of T, B, and NK cells (126).

### Genetic Engineering of DCs

We and others have used gene transfer technology to generate tolDCs *via* increased expression of immunomodulatory molecules such as IL-10, TGF-β, CTLA-4, IDO, PD-L1, or ligands for receptors resulting in T-cell deletion such as CD95/Fas (TNF-family related death receptor) and TNF-related apoptosis-inducing ligand (127–129). Using gene therapy approaches, recombinant adenovirus vectors typically achieve great transfection efficiencies but are limited by pro-inflammatory effects leading to DCs maturation (130). Methods using recombinant retrovirus vectors and, recently, non-viral gene transfer methods such as nucleoporation may induce lower degrees of DCs maturation (131). As an example, genetically engineered DCs over-expressing IDO regulated T-cell alloresponses *in vitro*, while IDO adenovirus-mediated gene transfer into the donor heart attenuated acute rejection of MHC-mismatched cardiac allografts in rats (132). Compared to pharmacological conditioning, genetic engineering of DCs using retroviral or lentiviral vectors offers the advantage of a potentially more stable cell phenotype and function *in vivo*. Another interesting approach that was described is the *in vivo* transfer of antigen-encoding bone marrow progenitor cells which, at steady-state, prevent antigen-specific sensitization and promote T-cell tolerance mostly by deletional mechanisms (133–135).

### DC Exposure to Apoptotic Cells

Exposure of DCs to early apoptotic cells down-modulates their stimulatory functions (136). DC internalization of apoptotic-cells-associated molecular patterns selectively leads to decreased production of pro-inflammatory cytokines (e.g., IL-1, TNF-α, IL-6, and IL-12), while enhancing the secretion of IL-10 and TGF-β (137). This process also limits upregulation of MHC class II and costimulatory molecules such as CD40, CD80 and CD86, hence maintaining DCs in an immature state (138). This mechanism contributes to self-tolerance and was exploited to induce tolerance to alloantigens in SOT. Following intravenous injection, donor allogeneic apoptotic cells were rapidly internalized in the spleen by red pulp macrophages and marginal zone DCs and were able to prolong cardiac allograft survival in rodents (139). Interestingly, T-cell depleting therapies used in clinical SOT, such as anti-CD3 or anti-CD52 monoclonal antibodies, were shown to induce T-cell apoptosis *in vivo*. This effect was associated with TGF-β secretion by DCs and subsequent expansion of Treg (140). Thus, administration of apoptotic cells or direct induction of apoptosis *in vivo* could be used to promote the generation of tolDCs *in vivo*. Of note, the induction of apoptosis *in vivo* must be carefully controlled to prevent simultaneous activation of the necrotic cell death mechanism and subsequent release of necrotic cell-associated antigens. These antigens were demonstrated to be able to stimulate CD141^+^CD14^−^ DCs through the Clec9A receptor favoring antigen cross-presentation and, hence, CD8^+^ T-cell responses (8).

## Therapeutic Use of tolDCs in Autoimmune Diseases

In Europe and North America, 5% of adults, of whom two-thirds are females, suffer from autoimmune diseases, the most prevalent pathologies being type 1 diabetes (T1D), psoriasis, RA, inflammatory bowel disease (IBD), and multiple sclerosis (MS).

### Type 1 Diabetes

Type 1 diabetes is due to a breakdown of self-tolerance and is mainly orchestrated by CD4^+^ and CD8^+^ autoreactive T cells that activate B cells, resulting in the production of autoantibodies specific for pancreatic islets β-cell antigens with progressive immune-mediated destruction of the β-cell mass and insulin insufficiency (141). Insulin treatment increases the life expectancy of T1D patients; however, it often fails to prevent T1D-associated cardiovascular and renal complications with increased morbidity and mortality. This opens the way to immune-based interventions aiming at restoring immune tolerance and preventing early T1D either by targeting autoreactive T cells (142) or by modifying the immunogenicity of DC. As T1D is a progressive and not a relapsing-remitting autoimmune disease, there is only a window of opportunity treatment period at the onset of the disease, in order to reinstitute self-tolerance and preserve the function of the existing β-cells mass.

In non-obese diabetic (NOD) mice, GM-CSF treatment prevented the development of diabetes primarily by inducing tolDCs and Treg (143). Another therapeutic option for T1D consists of transplantation of pancreatic islets or whole pancreas; however, such approaches require long-term immunosuppression to prevent allograft rejection and reoccurrence of autoimmunity. In an experimental model of syngeneic pancreatic islets transplantation in NOD mice, both transient TGF-β expression within islets and transplantation of islets grafts containing TGF-β-conditioned tolDCs reduced the activation of islet autoantigen-specific T cells in graft-draining LNs, resulting in prolonged graft survival (144). These results support the notion that TGF-β-induced tolDCs could be an effective strategy to restore peripheral tolerance within the context of an established autoimmune disease. Aside from generating tolDC, TGF-β promoted the survival of thymic-derived Treg and the differentiation of iTreg (64, 145, 146).

Other strategies using tolDCs for the prevention or treatment of T1D currently are being explored in experimental models. Antisense oligonucleotides have been used to specifically downregulate costimulatory molecules, resulting in DCs with an immature phenotype. A single injection of bone marrow-derived DCs engineered *ex vivo* with a mixture of antisense oligonucleotides targeting the CD40, CD80, and CD86 primary transcripts significantly delayed the onset of diabetes in syngeneic NOD recipients. The beneficial effect of these tolDCs was partly mediated by an increase in Treg (147). Based on these encouraging experimental data, efforts have been made at translating the use of autologous tolDCs in patients with new-onset T1D, aiming to prevent disease progression, or even to revert established disease (148, 149). A phase I clinical study showed that intradermal injection of autologous monocyte-derived costimulation-impaired tolDCs (10 × 10^6^ cells every 2 weeks for a total of 4 administrations), treated *ex vivo* with antisense oligonucleotides, was safe and well tolerated in patients with established T1D (150). A phase II follow-up clinical trial (ClinicalTrials.gov identifier NCT02354911) using DCs isolated from patients with recent-onset T1D is ongoing. Potential therapeutic success will be evaluated through the improvement of the glycemic control as evidence for a preserved β-cell mass. A similar clinical study using costimulation-impaired tolDCs is currently registered (identifier NCT01947569). This clinical trial includes a sequential open-label, phase-IB safety assessment and a randomized, double-blind, phase-IIA efficacy trial aiming at maintaining and improving residual β-cell function in new-onset T1D patients.

### Psoriasis

Psoriasis is a chronic inflammatory skin disease mainly characterized by abnormal keratinocyte proliferation and differentiation causing thickening of the epidermis (151). The psoriatic skin shows a prominent infiltration of neutrophils in the epidermis, together with macrophages, DCs, and T cells in the dermis. The successful use of cyclosporine A, a drug that inhibits early T-cell activation by blocking the TCR-downstream calcium–calcineurin pathway, highlights the role of T cells in the pathogenesis of the disease (152). Psoriasis has been associated with impaired Treg suppressive functions, resulting in overproduction of pro-inflammatory cytokines such as TNF-α and IFN-γ, as well as IL-17 and IL-22 produced by Th1 and Th17 effector cells, respectively (151, 153–155). The neuropeptide α-MSH is a well known mediator of skin pigmentation and has recently been shown to exert anti-inflammatory and immunomodulatory activities (106). Treatment with α-MSH ameliorated psoriasis-like skin inflammation, in part by suppressing the proliferation and effector function of Th17 cells. The beneficial effect of α-MSH was shown to be mediated by tolDCs and functional iTreg (107).

### Rheumatoid Arthritis

Rheumatoid arthritis, an autoimmune disease associated with chronic joint inflammation and destruction, is characterized by infiltration of innate immune cells (neutrophils, monocytes, NK cells and DCs) as well as T and B cells in the synovial compartment (156). The current treatment of RA includes immunosuppressive drugs such as corticosteroids, cytokine antagonists (anti-TNF-α), costimulation blockade (CTLA-4 Ig), and B-cell depleting monoclonal antibodies. More recently, the potential of DC-mediated immunomodulation for the treatment of RA was investigated. Clinical-grade tolDCs have been generated from monocytes of patients with RA by conditioning them *ex vivo* with VitD3 and dexamethasone. The resulting tolDCs exhibited reduced costimulatory molecules expression, low production of pro-inflammatory cytokines, and impaired capacity to stimulate antigen-specific T cells. Importantly, the phenotype and functional characteristics of tolDCs generated from RA patients were comparable to those generated from healthy individuals. These tolDCs remained stable in the absence of immunosuppressive drugs even after further challenge with pro-inflammatory mediators (157). Interestingly, the tolDCs exhibited high cell-surface expression of TLR2 compared to mature immunogenic DC. The use of this marker should be considered in future immunotherapeutic protocols to assess the quality and stability of tolDCs produced *ex vivo*. An ongoing registered phase I randomized, placebo-controlled trial (identifier NCT01352858, AutoDECRA) aims to generate autologous tolDCs to be injected (single dose) into the knee joint of patients suffering from RA. This clinical study will evaluate the effect of injected tolDCs on both the local and the systemic disease activity.

### Inflammatory Bowel Disease

The gut mucosa is constantly exposed to food antigens, pathogens, and commensal microorganisms, and holds the largest mass of lymphoid tissues in the body. The interplay between the intestinal epithelium and the local innate and adaptive immune system is crucial to the maintenance of immune homeostasis and oral tolerance. IBD (Crohn’s disease and ulcerative colitis) is mainly a consequence of loss of peripheral tolerance to otherwise harmless bacterial flora with dysregulated T-cell function in response to local intestinal antigens (158). Current treatments include corticosteroids, azathioprine, and 6-mercaptopurine, as well as anti-TNF-α and anti-α_4_β_7_ integrin therapies in severe cases. T-cell activation and effector function is dependent on the microenvironment in which antigen presentation occurs; hence, tolDCs may have a potential for reestablishing intestinal immune regulation (159). CD103^+^ DCs are found in the human gut under normal conditions (160). These cells express low levels of CD40, TLR2 and TLR4, secrete IL-10 but not IL-12, and produce retinoic acid and IDO that promote the differentiation of iTreg and local immune regulation (49, 161). Peripheral tissue-resident pDCs exert a pivotal role in oral and mucosal tolerance (15, 16). An aberrant pDC distribution and effector function was described in the mesenteric LNs and inflamed mucosa of patients with Crohn’s disease compared to healthy individuals (162). Moreover, immature peripheral blood pDCs and cDCs were reduced during flares in IBD patients (163). A phase I randomized clinical study (identifier NCT02622763 TolDecCDintra) currently evaluates the safety and clinical efficacy of autologous tolerogenic ModDCs injected into the intestinal lesions identified by endoscopy in patients with refractory Crohn’s disease.

G-CSF therapy has been shown to be beneficial in Crohn’s disease patients. The benefit was associated with increased numbers of pDCs in the gut mucosa and induction of IL-10 production (164). IL-10 is a crucial immunoregulatory cytokine in the gut, as documented by severe spontaneous IBD in IL-10-knockout mice (165). Protocols are under development to produce tolDCs under clinical-grade conditions for IBD patients by conditioning ModDCs with IL-10, together with a cocktail of other cytokines and prostaglandin E_2_ (166). The generated tolDCs display a semi-mature phenotype (intermediate expression of CD80 and CD86, MHC class II low), produce IL-10 with low levels of IL-12p70, IL-23, and TNF-α, and remain stable even in pro-inflammatory conditions. These data suggest that the strategy based on using autologous DCs (derived from patients with autoimmune diseases) may indeed be feasible for future immune therapies. Although the initial monocyte population is selected from patients with an overt inflammatory disease, the cells can be conditioned to acquire beneficial tolerogenic properties *ex vivo*.

### Multiple Sclerosis

Multiple sclerosis, a chronic inflammatory disease of the central nervous system (CNS), is predominantly a T-cell-mediated autoimmune disease characterized by leukocyte infiltration into the CNS, demyelination, and axonal loss. Besides current strategies targeting T and/or B cells, tolDCs may represent a potential therapeutic approach. Myelin peptide-loaded tolDCs were generated *ex vivo*, exhibiting a stable semi-mature phenotype and an anti-inflammatory cytokine profile. These tolDCs induced antigen-specific hyporesponsiveness in myelin-reactive T cells isolated from relapsing-remitting MS patients (167). IFN-β is an immunomodulatory agent used in the treatment of MS (168). Using healthy donors as well as MS patients PBMCs, it was shown that *in vitro* treatment with IFN-β enhanced PD-L1 expression on monocytes and DCs, inhibited antigen-specific CD4^+^ T-cell activation, and increased Treg numbers. In addition, serial *in vivo* measurements in MS patients before and 6 months after initiation of IFN-β therapy revealed a significant increase in PD-L1 mRNA (169). Sex hormones such as estrogens can modulate immune responses, contributing to the observed difference in the incidence of autoimmune diseases between males and females. Although the overall incidence of autoimmune diseases is higher in women compared with men, estrogens were protective in the experimental autoimmune encephalomyelitis (EAE) animal model of MS, even during pregnancy (170, 171). *In vivo* exposure to estriol (E3), a pregnancy-specific estrogen, induced tolDCs (E3 tolDC) characterized by increased expression of the inhibitory molecules PD-L1, PD-L2, B7-H3, and B7-H4, as well as mediators such as IL-10 and TGF-β, along with decreased expression of IL-12, IL-23, and IL-6. The transfer of E3 tolDCs to mice prior to active induction of EAE prevented the development of the disease. The protective effect was associated with immune deviation from pathogenic Th1/Th17 cells to a Th2 response (172). Two phase I clinical trials are currently registered on the ClinicalTrials.gov website, assessing the feasibility and safety of tolDCs loaded with myelin peptide in patients with MS (identifier NCT02618902 and NCT02903537). The TOLERVIT-MS (NCT02903537) trial involves VitD3-induced tolDCs used at increasing doses, starting from 5 × 10^6^ cells. Interestingly, different routes of administration will be explored for optimal efficacy, such as intravenous, intradermal or direct intranodal cell injection into cervical LNs.

## Therapeutic Potential of tolDCs in SOT

In the absence of adequate immunosuppression after SOT, the recognition of donor alloantigens by recipient T cells initiates a strong immune response leading to alloimmunization and allograft rejection. As T cells play a central role in alloresponses, immunosuppressive regimens have been historically developed to target T cells, whereas recent attention has also been devoted to the roles of B cells and alloantibodies (173). The development of immunosuppressive drugs has led to decreased rates of acute rejection after SOT, but their long-term administration is associated with side-effects including cardiovascular and renal toxicities, as well as infections and tumors (174, 175). Moreover, current regimens have limited effects on T- and B-cell memory responses and may interfere with the induction and expansion of donor-specific Treg (83, 176). Therefore, inducing sustained donor-specific tolerance with minimal drug exposure remains an important goal to improve long-term outcomes in transplantation medicine.

Compared to immune responses to autoantigens or pathogens, SOT constitutes a unique situation whereby DCs present antigens to alloreactive T cells through three different mechanisms: the direct, indirect and semi-direct pathways (177). Recipient T cells can be activated by donor antigens either as intact allogeneic MHC:peptide complexes presented by donor mature DCs that have migrated out of the allograft (direct allorecognition) or as donor-derived MHC:peptide complexes that have been processed and presented by recipient DCs (indirect allorecognition) (178, 179). The semi-direct pathway involves the presentation by recipient DCs of intact donor MHC:peptide complexes that have been captured from donor cell membranes or exosomes (177). Therefore, DCs from either donor or recipient origin can be considered for the development of immunotherapeutic protocols in SOT.

### Donor-Derived tolDC

Experimental models have illustrated the potential of donor-derived tolDCs to promote peripheral transplantation tolerance through the induction of donor-specific T-cell hyporesponsiveness, deletion, and/or regulation of alloreactive T cells (180–182). Costimulation-deficient tolDCs generated from donor bone marrow in the presence of GM-CSF, without IL-4, significantly prolonged the survival of MHC-mismatched heart allografts in mice when delivered (2 × 10^6^ cells intravenously) 1 week before transplantation. The effect was only partially antigen-specific, as third-party tolDCs also prolonged graft survival, albeit to a lesser extent (median graft survival time 22 vs. 16.5 days, respectively; vs. 9.5 days in control non-treated mice) (180). However, *in vivo* maturation of the injected tolDC occurred, as evidenced by upregulation of CD80 and CD86, partially explaining the limited efficacy of tolDCs *in vivo*. Based on these results, a newer approach evaluated donor-derived tolDCs generated in the presence of GM-CSF and TGF-β, delivered in conjunction with CD40–CD40L costimulation blockade 1 week before transplantation. This strategy resulted in extended allograft survival (181). In a preclinical non-human primate (NHP) model, the infusion of donor-derived tolerogenic ModDCs (3.5–10 × 10^6^ cells/kg) in combination with B7-CD28 costimulation blockade (CTLA-4 Ig given at day −7 and up to 8 weeks after transplantation) and rapamycin (started on day −2 and tapered over 6 months) significantly prolonged renal allograft survival (median graft survival time 113.5 vs. 39.5 days in controls) (86). In this study, tolDCs generated *in vitro* with VitD3 and IL-10 expressed low MHC class II and costimulatory molecules, high levels of PD-L1, and were resistant to inflammation-induced maturation. *Ex vivo* immune monitoring demonstrated regulation of donor-reactive memory T cells in tolDC-treated NHP compared to controls. Importantly, no adverse events, and particularly no significant allosensitization, were observed in the recipients.

Donor-derived tolDCs could also contribute to the induction of donor-specific central tolerance after SOT. A recent study evaluated the thymus-homing ability of DCs and their tolerogenic function. FMS-related tyrosine kinase 3 ligand (Flt3L) is a cytokine that synergizes with other growth factors to stimulate the proliferation and differentiation of hematopoietic progenitor cells. Flt3L-induced bone marrow-derived DCs (FLDCs), but not GM-CSF-induced DCs, were able to traffic to recipient thymus after systemic injection. Infusion of allogeneic donor-derived FLDCs induced clonal deletion of both CD4 and CD8 single-positive alloreactive thymocytes, leading to donor-specific central tolerance and prolonged survival of donor skin grafts (183).

### Recipient-Derived tolDCs

It should be emphasized that the use of recipient autologous DCs appears to be more feasible than that of donor DCs, as tolDCs can be prepared from peripheral blood of the recipient before SOT. Recipient tolDCs could be generated and stored while the patient is on the waiting list, and loaded with donor-derived antigens (HLA peptides, donor cell lysates) at the time of transplantation. Taking advantage of linked suppression mechanisms, it could be sufficient to generate recipient tolDCs expressing some but not all donor alloantigens (184). Importantly, the use of recipient DCs allows for the generation of tolDCs with the potential to induce indirect pathway CD4^+^ T-cell hyporesponsiveness and donor-specific Treg, while also controlling anti-donor humoral responses, which could have protective effects against chronic rejection (185–188). Recipient DCs pulsed with donor allopeptides and injected into the thymus in combination with one dose of anti-lymphocyte serum 7 days before transplantation induced donor-specific tolerance to cardiac (189) and pancreatic islets (190) allografts in rats. In an experimental model, the infusion of recipient DCs expressing donor intact MHC class I antigens selectively prevented indirect pathway alloreactive CD4^+^ T cells activation and the generation of alloantibodies, resulting in long-term survival of MHC-mismatched skin allografts (191). Interestingly, recipient bone marrow-derived tolDCs (generated in the presence of low-dose GM-CSF) infused one day before transplantation significantly prolonged cardiac allograft survival, even in the absence of prior *in vitro* pulsing with donor antigens. Although recipients showed reduced anti-donor cellular and humoral responses *ex vivo*, the effect of these tolDCs was not antigen-specific (192). This study suggests that, while alloantigen-specific immune regulation is desirable in the setting of SOT, recipient autologous tolDCs may also modulate the microenvironment in a way that favors allograft survival. The ONE study consortium aims at promoting clinical tolerance in living-donor renal transplant recipients (193). Within this consortium, a multicenter phase I/II safety/efficacy trial (ClinicalTrials.gov identifier NCT02252055) is currently evaluating the administration of autologous tolDCs on top of the standard immunosuppressive regimen (tacrolimus-MPA-corticosteroids).

## Feasibility and Safety of DC-Based Immunotherapy

### Phenotype and Function of Generated tolDCs

In recent years, an improved understanding of the mechanisms that govern central and peripheral immune tolerance, along with a more precise characterization of DC subsets, has opened the door to their therapeutic use in autoimmune diseases and SOT (Table 3). There are, however, some caveats that need to be addressed in order to safely translate and evaluate DC-based immunotherapy to the clinical arena. One major hurdle is the generation of high amounts of tolDCs of reproducible quality using clinical-grade procedures. Cells isolation, culture, and preconditioning protocols need to be optimized to generate tolDCs with stable phenotypes and functions. A better identification of subset-specific markers of human tolDCs would be valuable for safe delivery to patients. Another major concern is the stability of *ex vivo* generated tolDCs in a pro-inflammatory environment *in vivo*. While many studies support a maturation-resistant state of human tolDCs produced *in vitro* using various conditioning regimens, the *in vivo* environment may still induce maturation toward undesired immunogenic DCs (180, 194, 195). In the context of SOT, infused donor tolDCs have been shown to be short-lived, being eliminated mainly by recipient NK cells. Autologous recipient DCs could then process and present donor-derived MHC:peptide alloantigens in an immunogenic context, leading to allosensitization and accelerated allograft rejection (196). However, this aspect remains controversial as there is evidence suggesting that, while donor tolDCs indeed rapidly die after infusion and are re-processed by splenic recipient DC, this may not lead to allograft rejection because alloantigens are delivered in the context of tolDCs (197). Nonetheless, tolDC-based “negative vaccination” most likely will need to be combined with immunomodulatory drugs *in vivo* to harness strong immune activation, as demonstrated in NHP transplantation models (86, 198). Overall, additional preclinical and clinical trials are now needed to demonstrate the feasibility and safety of tolDC-based immunotherapy in humans. For instance, in a clinical study antigen-loaded autologous tolDCs were injected intradermic in healthy volunteers. While the treatment was well tolerated, it also resulted in antigen-specific regulation of Teff (199, 200).

**Table 3:** Tolerogenic DCs (tolDCs)-based clinical trials.

NCT identifier	Phase	Therapeutic agent	Status	Sponsor/collaborators	Disease
NCT00445913	I	Autologous dendritic cell (DC)	completed	University of Pittsburgh	T1DM
NCT02354911	II	Autologous immunoregulatory DC	Not yet recruiting	DiaVacs, Inc., and others	T1DM
NCT01947569	I/II	Autologous co-stimulation-impaired DC	Unknown	DiaVacs, Inc.	T1DM
NCT00434850	II	Deoxyspergualin, an immunosuppressant drug, shown to modulate DC differentiation and function	Completed	NIAID and NIDDK	Islets transplantation in T1DM
NCT01352858	I	Autologous tolDC	Unknown	Newcastle University and Arthritis Research UK	Rheumatoid arthritis (RA)
NCT00279461	II	Vit D3	Withdrawn	Indiana University	RA
NCT02283671	I	tolDCs loaded with myelin peptides	Currently recruiting	Sara Varea	Multiple Sclerosis (MS) and Neuromyelitis Optica
NCT02618902	I	tolDCs	Not yet recruiting	University Hospital, Antwerp	MS
NCT02903537	I	Autologous tolerogenic modDCs loaded with a pool of myelin peptides (tolDC-VitD3)	Not yet recruiting	Fundació Institut Germans Trias i Pujol	MS
NCT02622763	I	Intralesional administration of tolDCs	Currently recruiting	Fundacion Clinic per a la Recerca Biomédica	Crohn’s Disease
NCT02252055	I/II	Autologous tolDCs	Currently recruiting	Nantes University Hospital	Kidney transplantation

*NP, not provided; T1DM, type 1 diabetes mellitus; NIAID, National Institute of Allergy and Infectious Diseases; NIDDK, National Institute of Diabetes and Digestive and Kidney Diseases*.

### Source of DCs and Administration Protocols

The source of DCs is a central aspect of tolDC-based strategies. In the context of autoimmunity or end-stage organ disease, a possible effect of the disease on DCs population and function needs to be addressed. Specifically, it must be demonstrated that autologous tolDCs from patients are as stable as tolDCs derived from healthy individuals (157). The number of cells to be delivered, the most appropriate timing of injection, and the route of administration also need to be carefully evaluated. Antigen-specificity of DC-based immunomodulation is another open issue with respect to clinical applications. Targeted regulation of antigen-specific T-cell responses would avoid generalized immunosuppression and impaired immune surveillance leading to infections or the development of malignancies. However, specific autoantigens that are responsible for T-cell priming have not been identified in some autoimmune diseases such as IBD, and peripheral DCs used for immunotherapy may not present some tissue-specific antigens. It also should be considered that, in deceased donor SOT, donor alloantigens are not known until the day of transplantation.

In an attempt to limit the workload and costs of *ex vivo* generation of tolDCs, while also circumventing the uncertainty regarding their stability, these cells could be directly generated *in vivo*. As discussed, the administration of apoptotic cells or *in vivo* induction of apoptosis could be a first option in this regard (136, 201). Another possibility may involve the administration of specific immunomodulatory cytokines or drugs. Infliximab, a chimeric monoclonal antibody that neutralizes both soluble and membrane-bound TNF-α, is an effective treatment in autoimmune diseases such as psoriasis, RA and IBD, which can lead to long-term remission. Besides its inhibitory effects on T-cell activation and homing, infliximab was shown to impair the differentiation and antigen-presenting capacity of ModDCs (202) and to restore the suppressive function of previously compromised Treg in patients with RA (203). CTLA-4 Ig, a fusion protein that blocks B7-CD28 costimulation and is currently used in RA patients as well as in SOT, can also promote tolDC (204, 205). The combination of adoptive transfer of tolDCs and CTLA-4 Ig resulted in extended survival of MHC-mismatched heart allografts in mice (206). In an experimental model, CTLA-4 Ig suppressed collagen-induced arthritis by inducing tolDCs and expanding Treg. This effect was abrogated by anti-TGF-β treatment (207). Other drugs that can be used to modulate DCs functions are listed in Table 2. Finally, antigens could be directly delivered to steady-state quiescent DCs *in vivo* by specifically targeting DC-restricted endocytic receptors (DEC-205) with monoclonal antibodies (208), aiming at inducing antigen-specific T-cell hyporesponsiveness. Altogether, it will be important to take advantage of some currently used drugs or biological agents in order to promote an environment that favors tolDCs.

### Overcoming Memory Responses

Based on their central role in immune activation, it is very tempting to consider immunotherapy using tolDCs for tolerance induction to specific antigens. Indeed, the type of DC and the cytokine microenvironment at the time of antigen presentation are major components in the regulation of T-cell responsiveness. While many *in vitro* and *in vivo* experimental data support the potential of tolDCs in regulating the priming of naïve T cells, less evidence exist regarding memory T cells. This is an important issue as the human immune repertoire harbors antigen-specific as well as cross-reactive long-lived memory T cells and antibodies which could represent an obstacle for clinical translational of tolDCs-based immunotherapy in chronic autoimmune diseases as well as in SOT (209, 210). There are, however, some experimental and preclinical studies that illustrate the potential of tolDCs in controlling memory T cells (108, 133, 134), suggesting that tolDCs would be advantageous compared to Treg-based immunotherapy or more conventional immunosuppression that are less efficient in the presence of pre-existing memory responses (211). In an experimental allergic airway disease model, tolDCs were shown to inhibit allergen-specific memory Th2 responses and airway inflammation in sensitized hosts (135). In a clinically relevant NHP model of MHC-mismatched renal transplantation with minimal immunosuppression, infusion of donor-derived tolDCs 1 week before transplantation prolonged allograft survival, with no evidence of host sensitization. This therapeutic effect was associated with selective attenuation of donor-reactive memory T-cell responses (86, 198).

### Migration of tolDCs

Although significant progress has been made in the generation of tolDCs, the capacity of DCs to access LNs and encounter T cells, as well as the survival of treated DCs remains a critical concern. It is known that immature DCs traffic poorly to LNs. Different approaches have been made to overcome this issue. Genetically engineered DCs that express CCR7 and IL-10 showed improved migration ability to T-cell zones of secondary lymphoid organs and promoted prolonged heart allograft survival in a mouse model (212). Interestingly, in the setting of cancer vaccines development, the direct intra-lymphatic delivery approach is being evaluated in a clinical setting. DCs injected into a lymphatic vessel showed a prolonged half-life compared to DCs injected intravenously and were rapidly detected in LNs where they elicited a strong T-cell response (213, 214). The technique proved to be feasible and safe for short-term delivery of DCs. However, research is still needed to assess the efficacy of this route and the clinical relevance in the context of tolerance induction.

## Conclusion and Perspectives

The field of immune regulation has become increasingly complex with the identification of both lymphoid and non-lymphoid cells that are involved in the induction and maintenance of immune tolerance. DCs are at the cornerstone of adaptive immune responses and, therefore, represent an appealing tool for immunoregulatory therapies in autoimmune diseases as well as in SOT. While extensive data have been generated in animal models, questions remain regarding the distribution and function of human DCs subsets *in vivo*. Clinical protocols need to be optimized for the safe generation of tolDCs, ensuring stable phenotypes and immunomodulatory functions. Interestingly, although both exogenously transferred or *in vivo* induced tolDCs may have a short half-life, they were shown to induce a regulatory environment (regulatory cell-subsets and cytokines) that could promote a more prolonged maintenance of peripheral tolerance (215). Finally, tolDCs immunotherapy has a unique potential for inducing antigen-specific central tolerance.

Whether or not tolDCs can maintain their function and regulate memory T-cell and B-cell responses in a strong inflammatory environment still remains to be demonstrated in more stringent preclinical models (209, 216, 217). In the clinical setting, tolDC-based therapy, therefore, may not be sufficient *per se* to promote tolerance and may need to be used in conjunction with immunomodulatory drugs. While further preclinical and clinical studies are needed before tolDC-based immunotherapy can be successfully translated to the clinic, the quest for modalities to induce immune tolerance has clearly improved our understanding of human DC biology. The production and use of tolDCs in the upcoming years will continue to represent a challenging and exciting road, which, ultimately, may improve various immune-mediated clinical pathologies.

## Author Contributions

All authors contributed to writing the article. The final version of the manuscript was reviewed and approved by all authors.

## Conflict of Interest Statement

The authors declare that the research was conducted in the absence of any commercial or financial relationships that could be construed as a potential conflict of interest.
